# Clinical Testing for COVID-19, Influenza, and RSV in Hospitalized Youths, 2016-2024

**DOI:** 10.1001/jamanetworkopen.2025.31499

**Published:** 2025-09-15

**Authors:** Ariana P. Toepfer, Rachel E. Rutkowski, Leila C. Sahni, Geoffrey A. Weinberg, Marian G. Michaels, Rangaraj Selvarangan, Mary Allen Staat, Natasha Halasa, Janet A. Englund, Julie A. Boom, Peter G. Szilagyi, Jennifer E. Schuster, Elizabeth P. Schlaudecker, Laura S. Stewart, Eileen J. Klein, Samantha M. Olson, Sascha Ellington, Meredith L. McMorrow, Heidi L. Moline, Fatimah S. Dawood

**Affiliations:** 1 National Center for Immunization and Respiratory Diseases, Centers for Disease Control and Prevention, Atlanta, Georgia; 2 US Public Health Service, Rockville, Maryland; 3 Texas Children’s Hospital, Houston; 4 Department of Pediatrics, Baylor College of Medicine; 5 Department of Pediatrics, University of Rochester School of Medicine and Dentistry, Rochester, New York; 6 Department of Pediatrics University of Pittsburgh School of Medicine (UPMC), UPMC Children’s Hospital of Pittsburgh, Pittsburgh, Pennsylvania; 7 Children’s Mercy, Kansas City, Missouri; 8 Department of Pediatrics, University of Cincinnati College of Medicine, Division of Infectious Diseases, Cincinnati Children’s Hospital Medical Center, Cincinnati, Ohio; 9 Vanderbilt University Medical Center, Nashville, Tennessee; 10 Seattle Children’s Research Institute, Department of Pediatrics, University of Washington, Seattle; 11 Department of Pediatrics, University of California at Los Angeles

## Abstract

**Importance:**

Studies of COVID-19, influenza, and respiratory syncytial virus (RSV) disease clinical epidemiology, disease burden, and vaccine effectiveness often rely on clinical testing to identify cases, particularly with increasing use of electronic health record data. However, few data are available describing clinical testing practices to inform interpretation of these studies.

**Objective:**

To characterize clinical testing frequency for SARS-CoV-2, influenza, and RSV among children and adolescents hospitalized with acute respiratory illness during the 2016 to 2024 respiratory illness seasons by age, intensive care unit admission, and time period (2016-2020 and each season in 2020-2024); estimate the case fraction missed by clinical testing alone; and identify patient characteristics associated with clinical testing.

**Design, Setting, and Participants:**

This cross-sectional study analyzed data collected through prospective surveillance with systematic molecular testing (surveillance testing) for SARS-CoV-2, influenza viruses, and RSV among children and adolescents younger than 18 years hospitalized with acute respiratory illness at 6 US medical centers during the winter respiratory illness seasons of 2016 to 2024. In-hospital clinical molecular testing data were abstracted from the medical record.

**Main Outcomes and Measures:**

The proportion of children and adolescents with clinical molecular testing for SARS-CoV-2, influenza viruses, and RSV.

**Results:**

During 2016 to 2024, a total of 26 073 hospitalized youths were enrolled (14 459 aged <2 years [55.5%]; 14 833 male [56.9%]), including 12 090 youths during 2016 to 2020 combined. Overall, 10 450 youths (40.1%) had an underlying medical condition. The proportion of youths with clinical testing decreased for SARS-CoV-2 from 3965 of 4388 youths (90.4%) during 2020 to 2021 to 2802 of 3685 youths (76.0%) during 2023 to 2024 (*P* < .001). In contrast, clinical testing increased from the 2016 to 2020 seasons to the 2023 to 2024 season for influenza viruses (5384 of 12 090 youths [44.5%] to 2740 of 3685 youths [74.4%]; *P* < .001) and RSV (5072 youths [42.0%] to 2638 youths [71.6%]; *P* < .001). During the 2023 to 2024 season, the proportion of youths identified as positive for each virus by surveillance testing alone (ie, without clinical testing) was 23 of 112 youths (20.5%) for SARS-CoV-2, 78 of 286 youths (27.3%) for influenza, and 290 of 979 youths (29.6%) for RSV; youths were more likely to receive clinical testing if they were admitted to the intensive care unit (eg, SARS-CoV-2: adjusted odds ratio [aOR], 2.62; 95% CI, 2.06-3.33) or had underlying conditions (eg, any vs no underlying condition for SARS-CoV-2: aOR, 1.56; 95% CI, 1.33-1.84).

**Conclusions and Relevance:**

In this study, SARS-CoV-2 clinical testing decreased between 2020 to 2021 and 2023 to 2024 seasons, whereas influenza and RSV testing increased significantly between 2016 to 2020 and 2023 to 2024 seasons; approximately three-quarters of children and adolescents were tested for the 3 viruses in 2023 to 2024.

## Key Points

**Question:**

Did clinical testing practices for COVID-19, influenza, and respiratory syncytial virus (RSV) change over time during 2016 to 2024 among hospitalized children and adolescents?

**Findings:**

In this cross-sectional study among 26 073 children and adolescents, clinical testing for influenza and RSV increased from 2016 to 2024, with approximately three-quarters of all youths tested in 2023 to 2024, whereas SARS-CoV-2 clinical testing decreased after 2020.

**Meaning:**

These findings suggest that clinical testing practices may continue to evolve over time and that understanding testing patterns is critical to interpreting the increasing number of studies that rely on clinical testing for case identification.

## Teaser

This cross-sectional study investigates trends in frequency of clinical testing for COVID-19, influenza, and respiratory syncytial virus from 2016 to 2024 among hospitalized youths with acute respiratory illness.

## Introduction

Acute respiratory illnesses (ARIs) are a leading cause of hospitalization in young children. Respiratory syncytial virus (RSV) and influenza viruses are recognized as common causes of ARI hospitalization in children and adolescents during the winter respiratory illness season.^1,2,3,4^ Since the emergence of SARS-CoV-2, the virus that causes COVID-19, this illness has also contributed to annual ARI hospitalization burden in children and adolescents.^5,6^ Clinical testing for SARS-CoV-2, influenza, and RSV among hospitalized children and adolescents with ARIs can inform hospital infection-control practices and clinical diagnosis and management,^7,8^ but clinical testing practices may vary by hospital and by patient characteristics.

Studies that characterize COVID-19, influenza, and RSV disease clinical epidemiology, disease burden, and vaccine effectiveness often rely on clinical testing to identify cases, particularly with the increasing use of electronic health record (EHR) data. Understanding clinical testing practices can inform interpretation of these studies by estimating the potential magnitude of underascertainment for burden studies and identifying potential biases in clinical testing that impact study representativeness.^9^ Additionally, identifying longitudinal variation in clinical testing by pathogen can aid in interpretation of comparative and longitudinal analyses based on clinical testing, which may otherwise lead to incorrect conclusions about relative burden and time trends.

A systematic review^10^ summarized clinical testing practices for RSV among children younger than 5 years during 1988 to 2020 and found only 4 studies among hospitalized children. An EHR-based study published in 2024^11^ found that RSV clinical testing practices among infants changed during the COVID-19 pandemic but did not evaluate clinical testing practices for COVID-19 or influenza. To date, few data exist examining trends in clinical testing and detection of SARS-CoV-2, influenza viruses, and RSV among hospitalized children and adolescents to assess how testing practices may differ by virus type and whether testing practices changed during and after the emergence of SARS-CoV-2.

The New Vaccine Surveillance Network (NVSN) is a prospective, population-based surveillance network that conducts systematic molecular testing for SARS-CoV-2, influenza, and RSV among children and adolescents hospitalized with ARI. We used data from NVSN during the winter respiratory illness seasons of 2016 to 2024 (October 1 to April 30) to characterize the frequency of clinical testing for SARS-CoV-2, influenza, and RSV among children and adolescents hospitalized with ARIs by age, care setting (ie, intensive care unit [ICU]), and time period and estimated the proportion of individuals with illness who were missed when relying on only clinical testing for case identification. For the 2023 to 2024 season, we also examined patient characteristics and severity markers associated with clinical testing to identify potential biases in case detection.

## Methods

### Setting and Study Design

Data for this cross-sectional study were collected from 6 pediatric academic medical centers, in Cincinnati, Ohio; Houston, Texas; Kansas City, Missouri; Nashville, Tennessee; Pittsburgh, Pennsylvania; and Rochester, New York. During December 1, 2016, to April 30, 2024, NVSN enrolled children and adolescents younger than 18 years hospitalized with ARIs with illness onset within the past 14 days (NVSN ARI inclusion criteria changed in November 2022 to include children and adolescents with an illness onset within 10 days instead of 14 days of hospitalization). ARI was defined as at least 1 of the following: apnea, cough, earache, fever, myalgia, nasal congestion, runny nose, sore throat, vomiting after coughing, shortness of breath, wheezing, or apparent life-threatening event or brief resolved unexplained event. Data on demographic and clinical characteristics were collected through parent or guardian interviews and EHR reviews, as previously described.^6,12^ Race and ethnicity were based on parent report. Race and ethnicity categories were selected based on the Office of Management and Budget Standards for Maintaining, Collecting, and Presenting Federal Data on Race and Ethnicity and were reported to characterize the representativeness of the analytic population. Race options on surveys included American Indian or Alaska Native, Asian, Black or African American, Native Hawaiian or Pacific Islander, White, and other. Ethnicity options on surveys included Hispanic and not Hispanic. Data on molecular-based reverse transcriptase–polymerase chain reaction (RT-PCR) clinical testing were collected from the medical record. Clinical testing assays used in hospital differed by site and season (eTable 3 in Supplement 1).

### Ethics

During 2016 to 2022, written informed consent was obtained from a parent or legal guardian for all youths prior to any data or surveillance specimen collection. Assent from eligible youths was obtained at each site according to local regulations. Study activities during 2016 to 2020 were reviewed and approved by institutional review boards at the Centers for Disease Control and Prevention (CDC) and each site. Study activities during 2023 to 2024 were reviewed by the CDC, were deemed not research, and were conducted consistent with applicable federal law and CDC policy.^13,14^ This study is reported according to the Strengthening the Reporting of Observational Studies in Epidemiology (STROBE) reporting guideline.

### Laboratory Testing

Enrolled youths had midturbinate nasal or throat swabs or both collected for SARS-CoV-2, influenza, and RSV surveillance testing by RT-PCR using assays that met CDC proficiency testing standards. For patients from whom research specimens could not be obtained, clinically obtained respiratory specimens were salvaged for surveillance testing.^6^

### Statistical Analysis

This cross-sectional analysis included hospitalized youths enrolled in NVSN who had information available about clinical testing and had surveillance testing completed. The analytic period was defined as October 1 to April 30 (winter respiratory illness season) of 2016 to 2024, with the exception of the 2016 to 2017 and 2020 to 2021 seasons. The 2016 to 2017 season began on December 1, when NVSN data collection began. The 2020 to 2021 season was defined as October 1, 2020, to September 30, 2021, because of an absence of influenza virus circulation and atypical summer RSV circulation in 2021. For 2016 to 2020 seasons, only testing for influenza and RSV was evaluated. Testing for SARS-CoV-2 was evaluated starting with the 2020 to 2021 season because systematic testing for SARS-CoV-2 began in April 2020.

The proportion of hospitalized youths with ARIs who received molecular testing for each virus as part of routine clinical care (ie, clinical testing) was summarized with frequencies and percentages by time period: 2016 to 2020 seasons combined and 2020 to 2021, 2022 to 2023, and 2023 to 2024 seasons separately. For 2016 to 2020 seasons, the proportion of hospitalized children and adolescents with clinical testing was first evaluated separately by season to confirm that proportions were stable over time before aggregating results for the 4 seasons (eTable 2 in Supplement 1). Results were also stratified by age group (0-2 months, 3-5 months, 6-11 months, 12-23 months, 24-59 months, 5-9 years, and 10-17 years) and clinical care setting. For RSV, clinical testing proportions were also examined by period among children younger than 2 years with a discharge diagnosis of bronchiolitis (defined as *International Classification of Diseases, Ninth Revision *[*ICD-9*] or *International Statistical Classification of Diseases and Related Health Problems, Tenth Revision *[*ICD-10*] code J21).^15^

Time trends in clinical testing for each pathogen overall, by age group, and by ICU status were assessed using the Cochran-Armitage test for trends. To account for unequal lengths of periods, midpoints for each period were used in trend testing. To assess the proportion of virus detections missed when relying solely on clinical testing for case detection (eg, those positive by surveillance testing but not clinically tested for the virus), youths were also stratified by clinical testing status and surveillance test results for each virus.

To examine factors associated with clinical molecular testing, separate multivariable logistic regression models were used to estimate the odds of clinical molecular testing among youths with each demographic and clinical characteristic of interest compared with specified reference groups, with adjustment for study site. Age, race and ethnicity, and insurance status were each categorized as individual variables, with all subgroups included in a single model. Analyses were restricted to the 2023 to 2024 season to focus on the most recent testing patterns. When assessing odds of clinical testing among youths with specific underlying conditions, youths without any underlying conditions were the comparison group.

*P* values were 2-sided, and a *P* value < .05 was considered statistically significant. All analyses were performed using SAS statistical software version 9.4 (SAS Institute).

## Results

### Analysis Population

During the 2016 to 2024 seasons, 26 073 hospitalized youths were enrolled (14 459 aged <2 years [55.5%]; 14 833 male [56.9%]; 626 Asian non-Hispanic [2.4%], 5981 Black non-Hispanic [22.9%], 6860 Hispanic [26.3%], 10 725 White non-Hispanic [41.1%], and 1303 multiple races or other nonspecified, non-Hispanic [5.0%]), including 12 090 youths during the 2016 to 2020 seasons combined and 2644 to 4388 youths per season during 2020 to 2024 (Table 1). Overall, 16 248 youths (62.3%) had public insurance and 10 450 youths (40.1%) had an underlying medical condition. Of 14 454 enrolled children younger than 2 years with discharge information, 5384 children (37.3%) had a discharge diagnosis of bronchiolitis (Table 1). Patient characteristics were similar by period from the 2016 to 2020 seasons through the 2023 to 2024 season.

**Table 1.** Study Population Characteristics

Characteristic	Youths with ARIs, No. (%) (N = 26 073)^a^					
	All	Winter ARI season^b^				
		2016-2020	2020-2021	2021-2022	2022-2023	2023-2024
Overall	26 073 (100)	12 090 (100)	4388 (100)	2644 (100)	3266 (100)	3685 (100)
Age group						
0-2 mo	4185 (16.1)	2150 (17.8)	707 (16.1)	338 (12.8)	482 (14.8)	508 (13.8)
3-5 mo	2274 (8.7)	1162 (9.6)	332 (7.6)	184 (7.0)	307 (9.4)	289 (7.8)
6-11 mo	3274 (12.6)	1594 (13.2)	542 (12.4)	257 (9.7)	413 (12.7)	468 (12.7)
12-23 mo	4726 (18.1)	2193 (18.1)	796 (18.1)	557 (21.1)	574 (17.6)	606 (16.5)
24-59 mo	5303 (20.3)	2270 (18.8)	866 (19.7)	640 (24.2)	763 (23.4)	764 (20.7)
5-9 y	3662 (14.0)	1600 (13.2)	538 (12.3)	385 (14.6)	478 (14.6)	661 (17.9)
10-17 y	2649 (10.2)	1121 (9.3)	607 (13.8)	283 (10.7)	249 (7.6)	389 (10.6)
Sex						
Male	14 833 (56.9)	6938 (57.4)	2463 (56.1)	1487 (56.2)	1883 (57.7)	2062 (56.0)
Female	11 240 (43.1)	5152 (42.6)	1925 (43.9)	1157 (43.8)	1383 (42.4)	1623 (44.0)
Race and ethnicity						
American Indian or Alaska Native, non-Hispanic	159 (0.6)	85 (0.7)	14 (0.3)	19 (0.7)	22 (0.7)	19 (0.5)
Asian, non-Hispanic	626 (2.4)	264 (2.2)	89 (2.0)	78 (3.0)	88 (2.7)	107 (2.9)
Black, non-Hispanic	5981 (22.9)	2916 (24.1)	1033 (23.5)	571 (21.6)	647 (19.8)	814 (22.1)
Hispanic or Latino	6860 (26.3)	2908 (24.1)	1231 (28.1)	681 (25.8)	1042 (31.9)	998 (27.1)
Native Hawaiian or Other Pacific Islander, non-Hispanic	83 (0.3)	37 (0.3)	16 (0.4)	6 (0.2)	12 (0.4)	12 (0.3)
White, non-Hispanic	10 725 (41.1)	5161 (42.7)	1660 (37.8)	1092 (41.3)	1294 (39.6)	1518 (41.2)
Multiple races or other nonspecified, non-Hispanic	1303 (5.0)	634 (5.2)	217 (5.0)	130 (4.9)	130 (4.0)	192 (5.2)
Unknown	336 (1.3)	85 (0.7)	128 (2.9)	67 (2.5)	31 (1.0)	25 (0.7)
Insurance						
Public	16 248 (62.3)	7770 (64.3)	2778 (63.3)	1615 (61.1)	1964 (60.1)	2121 (57.6)
Private	7818 (30.0)	3416 (28.3)	1376 (31.4)	837 (31.7)	1024 (31.4)	1165 (31.6)
Public and private	301 (1.2)	154 (1.3)	41 (0.9)	38 (1.4)	24 (0.7)	44 (1.2)
Self-pay (none)	1277 (4.9)	609 (5.0)	146 (3.3)	115 (4.4)	146 (4.5)	261 (7.1)
Any underlying condition	10 450 (40.1)	4880 (40.4)	1750 (39.9)	1010 (38.2)	1215 (37.2)	1595 (43.3)
Discharge diagnosis of bronchiolitis^c^^,^^d^	5384/14 454 (37.3)	2963/7099 (41.7)	731/2377 (30.8)	401/1336 (30.0)	637/1776 (35.9)	652/1866 (34.9)
Clinical testing^e^						
SARS-CoV-2^c^	11 708/13 983 (83.7)	NA	3965 (90.4)	2305 (87.2)	2636 (80.7)	2802 (76.0)
Influenza	14 923 (57.2)	5384 (44.5)	2436 (55.5)	1783 (67.4)	2580 (79.0)	2740 (74.4)
RSV	14 325 (54.9)	5072 (42.0)	2442 (55.7)	1718 (65.0)	2455 (75.2)	2638 (71.6)

Abbreviations: ARI, acute respiratory illness; NA, not applicable; RSV, respiratory syncytial virus.

^a^ ARI was defined as at least 1 of the following: apnea, cough, earache, fever, myalgia, nasal congestion, runny nose, sore throat, vomiting after coughing, shortness of breath, wheezing, or apparent life-threatening event or brief, unexplained but resolved event.

^b^ Periods were defined as October 1 to April 30 (respiratory season) of 2016 to 2024. Exceptions were the 2016 to 2017 season, which began on December 1, when New Vaccine Surveillance Network data collection began, and the 2020 to 2021 season, which was defined as October 1, 2020, to September 30, 2021, for analyses because of atypical RSV circulation and ongoing SARS-CoV-2 circulation during the summer months of 2021. The 2016 to 2020 seasons were aggregated, and seasons from 2020 to 2021 onward were analyzed individually.

^c^ Denominators are indicated in the table for discharge diagnosis of bronchiolitis and clinical testing for SARS-CoV-2 because the denominator differs from the overall denominator for these variables owing to variable-specific restrictions.

^d^ Bronchiolitis at discharge denominator is among children and infants younger than 2 years.

^e^ Clinical testing was defined as anyone who received a reverse transcriptase–polymerase chain reaction test for the virus of interest during the hospital visit.

### Clinical Testing Trends by Season

**Figure 1.**
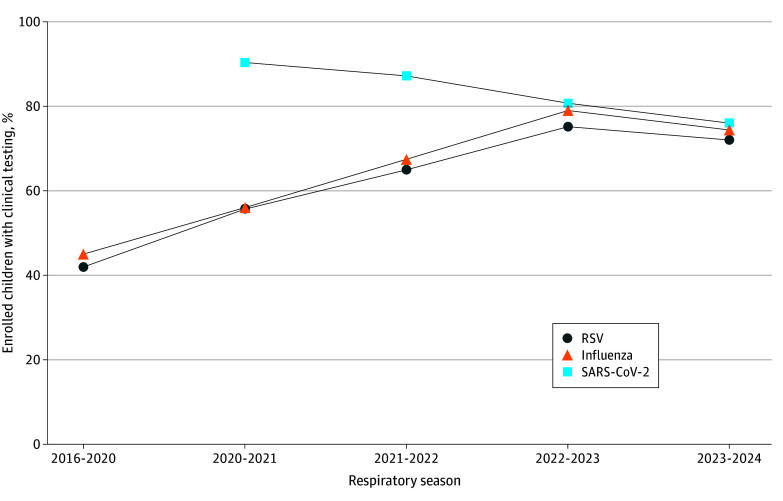
Trends in Clinical Testing Overall The Cochran-Armitage test for trend was statistically significant (*P* < .001) for respiratory syncytial virus (RSV), influenza, and SARS-CoV-2 trends over time. Midpoints of each period were used to represent time in trend testing to account for unequal spacing. Periods were defined as October 1 to April 30 (respiratory season) of 2016 to 2024. Exceptions were the 2016 to 2017 season, which began on December 1, when New Vaccine Surveillance Network data collection began, and the 2020 to 2021 season, which was defined as October 1, 2020, to September 30, 2021, for analyses because of atypical RSV circulation and ongoing SARS-CoV-2 circulation during the summer months of 2021. The 2016 to 2020 seasons were aggregated, and seasons from 2020 to 2021 onward were analyzed individually.

**Figure 2.**
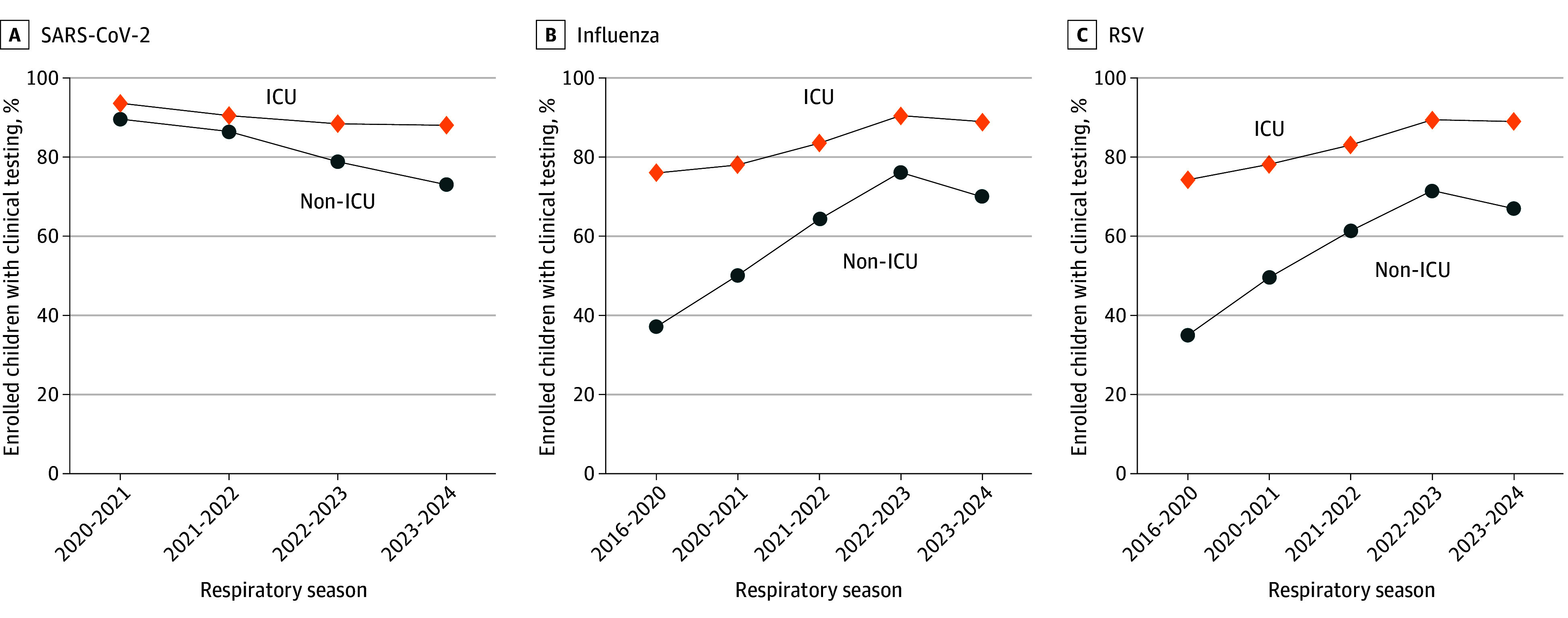
Trends in Clinical Testing by Care Setting The Cochran-Armitage test for trends was statistically significant for both intensive care unit (ICU) status groupings (*P* < .001). Midpoints of each period were used to represent time in trend testing to account for unequal spacing. Periods were defined as October 1 to April 30 (respiratory season) of 2016 to 2024. Exceptions were the 2016 to 2017 season, which began on December 1, when New Vaccine Surveillance Network data collection began, and the 2020 to 2021 season, which was defined as October 1, 2020, to September 30, 2021, for analyses because of atypical respiratory syncytial virus (RSV) circulation and ongoing SARS-CoV-2 circulation during the summer months of 2021. The 2016 to 2020 seasons were aggregated, and seasons from 2020 to 2021 onward were analyzed individually.

Overall, among 13 983 youths enrolled during 2020 to 2024, there were 11 708 youths (83.7%) tested for SARS-CoV-2, while among 26 073 youths enrolled during 2016 to 2024, there were 14 923 youths (57.2%) tested for influenza viruses and 14 325 youths (54.9%) tested for RSV (Table 1). The proportion of youths who received clinical testing decreased over time for SARS-CoV-2, from 3965 of 4388 youths (90.4%) during 2020 to 2021 to 2802 of 3685 youths (76.0%) during 2023 to 2024 (*P* < .001) (Figure 1). In contrast, the proportion of youths with clinical testing increased from the 2016 to 2020 seasons to the most recent season (2023-2024) for influenza viruses, from 5384 of 12 090 youths (44.5%) to 2740 of 3685 youths (74.4%) (*P* < .001), and for RSV, from 5072 youths (42.0%) to 2638 youths (71.6%), a 1.7-fold increase for each virus (*P* < .001) (Figure 1). For each virus, similar time trends in clinical testing were observed by age group (eFigure 3 in Supplement 1) and clinical care setting (Figure 2A-C).

### Clinical Testing by Age Group and Care Setting

By age group, the proportion of youths with clinical testing for SARS-CoV-2 was similar during each season from 2020 to 2021 (range by age group, 1478 of 1670 infants and children aged 3-23 months [88.5%] to 1051 of 1145 youths aged 5-17 years [91.8%]) to 2023 to 2024 (range by age group, 777 of 1050 youths aged 5-17 years [74.0%] to 596 of 764 youths aged 24-59 months [78.0%]) (eFigure 3 in Supplement 1). In contrast, the proportion of youths with clinical testing for influenza was consistently highest among infants aged 0 to 2 months during most seasons (range, 1086 of 2150 infants [50.5%] during 2016-2020 to 410 of 482 infants [85.1%] during 2022-2023), but the variation in testing proportions between age groups decreased over time (eFigure 3 in Supplement 1). A similar pattern was observed for clinical testing for RSV, with the highest testing proportion among infants aged 0 to 2 months during all time periods (range, 1064 of 2150 infants [49.5%] during 2016-2020 to 403 of 482 infants [83.6%] during the 2022-2023 season), with decreased variation in testing proportion between age groups over time (eFigure 3 in Supplement 1).

By care setting (non-ICU vs ICU hospitalization), the proportion of youths tested for SARS-CoV-2 was similar during the 2020 to 2021 and 2021 to 2022 seasons but diverged during the 2022 to 2023 and 2023 to 2024 seasons as testing among youths with non-ICU hospitalizations decreased (Figure 2A). In contrast, the proportion of youths tested for influenza was consistently higher by season among youths with ICU hospitalizations compared with those with non-ICU hospitalizations, but fold differences in testing proportions between care settings decreased over time, from 2.0 times higher testing among youths in the ICU during 2016 to 2020 (1612 of 2118 youths [76.1%] vs 3769 of 9960 youths [37.8%]; *P* < .001) to 1.3 times higher testing (693 of 778 youths [89.1%] vs 2037 of 2896 youths [70.3%]; *P* < .001) during 2023 to 2024. Among youths with non-ICU admissions, clinical testing for influenza increased 1.9-fold from the 2016 to 2020 seasons to the 2023 to 2024 season (37.8% to 70.3%). A similar pattern was also observed for clinical testing for RSV, with 2.1-fold higher testing among youths with ICU hospitalizations compared with non-ICU hospitalizations (1573 of 2118 youths [74.3%] vs 3496 of 9960 youths [35.1%]; *P* < .001) during 2016 to 2020 to 1.3-fold higher (689 of 778 youths [88.6%] vs 1939 of 2896 youths [67.0%]; *P* < .001) during 2023 to 2024. Among youths with non-ICU admissions, clinical testing for RSV increased 1.9-fold from the 2016 to 2020 seasons to the 2023 to 2024 seasons (35.1% to 67.0%) (Figure 2A-C).

### Illnesses Missed by Clinical Testing and Percent Positivity by Clinical vs Surveillance Testing

During the 2020 to 2024 seasons, the proportion of enrolled youths per season who did not have clinical testing ranged from 423 of 4388 youths (9.6%) in 2020 to 2021 to 883 of 3685 youths (24.0%) in 2023 to 2024 for SARS-CoV-2, from 686 of 3266 youths (21.0%) in 2022 to 2023 to 1952 of 4388 youths in 2020 to 2021 (44.5%) for influenza, and from 811 of 3266 youths (24.8%) in 2022 to 2023 to 1946 of 4388 youths (44.3%) in 2020 to 2021 for RSV (Table 1). Of all youths with SARS-CoV-2, influenza, and RSV identified by NVSN systematic surveillance testing during 2020 to 2024, the proportion of infections identified by systematic surveillance testing alone ranged from 13 of 85 infections (15.6%) in 2022 to 2023 to 49 of 209 infections (23.4%) in 2021 to 2022 for COVID-19, from 1 of 6 infections (16.7%) in 2020 to 2021 to 78 of 286 infections (27.3%) in 2023 to 2024 for influenza, and from 214 of 844 infections (25.4%) in 2022 to 2023 to 374 of 917 infections (40.8%) in 2020 to 2021 for RSV disease (Table 2). For 2023 to 2024, the proportions identified solely by surveillance testing for SARS-CoV-2 and RSV were 23 of 122 youths (20.5%) and 290 of 979 youths (29.6%), respectively. The proportions of tests that were positive for each virus among youths who had clinical vs surveillance testing differed during some seasons (Table 2).

**Table 2.** Percent Positivity by Clinical Testing Status Among Youths With Systematic Surveillance Testing

Clinical testing status	Youths, No.				
	2016-2020^a^	2020-2021^a^	2021-2022^a^	2022-2023^a^	2023-2024^a^
**SARS-CoV-2**					
Total surveillance tests	NA	4033	2481	3083	3551
With clinical testing	NA	3633	2156	2483	2693
Positive in systematic surveillance, No. (%)	NA	190 (5.2)	160 (7.4)	72 (2.9)	89 (3.3)
Without clinical testing	NA	400	325	600	858
Positive in systematic surveillance, No. (%)	NA	35 (8.8)	49 (15.1)	13 (2.2)	23 (2.7)
**Influenza viruses**					
Total surveillance tests	11 920	4036	2481	3083	3551
With clinical testing	4474	1780	1654	2428	2638
Positive in systematic surveillance, No. (%)	335 (7.5)	5 (0.3)	83 (5.1)	136 (5.6)	208 (7.9)
Without clinical testing	7446	2256	827	655	913
Positive in systematic surveillance, No. (%)	658 (8.8)	1 (<0.1)	17 (2.1)	44 (6.7)	78 (8.5)
**RSV**					
Total surveillance tests	11 920	4036	2481	3083	3551
With clinical testing	4945	2187	1590	2308	2537
Positive in systematic surveillance, No. (%)	1511 (30.6)	543 (24.8)	232 (14.6)	630 (27.3)	689 (27.2)
Without clinical testing	6975	1849	891	775	1014
Positive in systematic surveillance, No. (%)	2259 (32.4)	374 (20.2)	89 (10.0)	214 (27.6)	290 (28.6)

Abbreviations: NA, not applicable; RSV, respiratory syncytial virus.

^a^ Periods were defined as October 1 to April 30 (respiratory season) of 2016 to 2024. Exceptions were the 2016 to 2017 season, which began on December 1, when New Vaccine Surveillance Network data collection began, and the 2020 to 2021 season, which was defined as October 1, 2020, to September 30, 2021, for analyses because of atypical RSV circulation and ongoing SARS-CoV-2 circulation during the summer months of 2021. The 2016 to 2020 seasons were aggregated, and seasons from 2020 to 2021 onward were analyzed individually.

### Characteristics Associated With Clinical Testing, 2023 to 2024 Season

**Figure 3.**
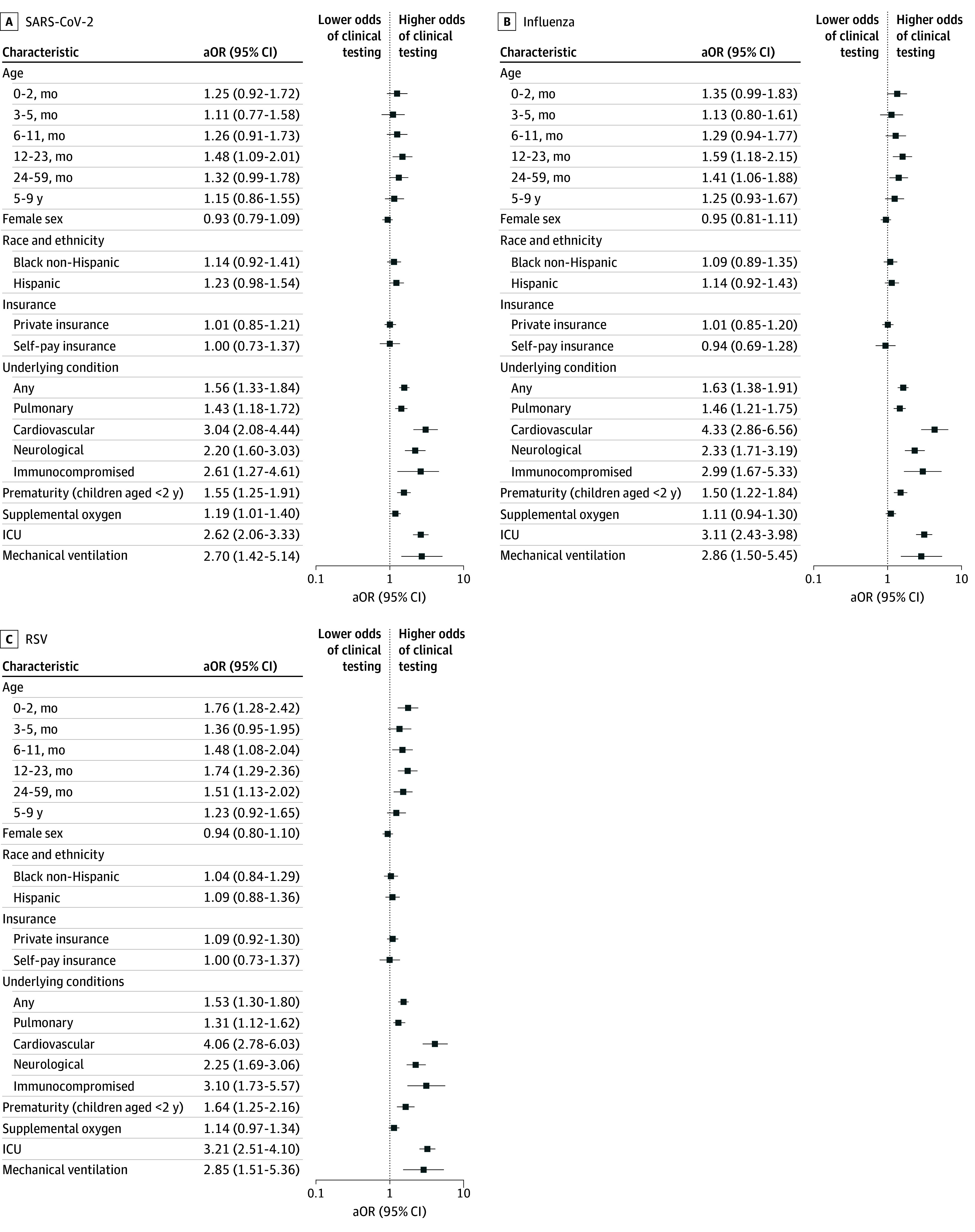
Association of Youth Characteristics With Clinical Testing, 2023-2024 All adjusted odds ratios (aORs) are derived from logistic models adjusted for study site. Reference groups for comparisons were ages 10 to 17 years for age, male sex for sex, White non-Hispanic for race and ethnicity, public insurance for insurance status, no underlying conditions for any underlying condition and specific underlying conditions, infants and children born at term aged younger than 2 years for prematurity, and no supplemental oxygen, intensive care unit (ICU) admission, or mechanical ventilation for each of these clinical outcomes, respectively.

Among 3685 youths enrolled during the 2023 to 2024 season, clinical testing was done among 2802 youths (76.0%) for SARS-CoV-2, among 2740 youths (74.4%) for influenza viruses, and among 2638 youths (71.6%) for RSV (Table 1). Compared with older children and adolescents (ages 10-17 years), children aged 12 to 23 months had higher odds of being tested for influenza and SARS-CoV-2, and infants and children aged 0 to 2 months, 6 to 11 months, 12 to 23 months, and 24 to 59 months had higher odds of being tested for RSV (Figure 3). There were no associations of sex, race and ethnicity, or insurance status with clinical testing for any virus (Figure 3). Youths with at least 1 underlying condition had higher odds of clinical testing than those without underlying conditions for all 3 viruses (eg, any underlying condition for SARS-CoV-2: adjusted odds ratio [aOR], 1.56; 95% CI, 1.33-1.84) (Figure 3). Similarly, youths with pulmonary, cardiovascular, neurologic or neuromuscular, or immunocompromising conditions had increased odds of clinical testing for all 3 viruses, with the exception of pulmonary conditions among youths tested for RSV. Children and infants younger than 2 years with prematurity also had increased odds of testing for the 3 pathogens, as did youths with ICU admission (eg, SARS-CoV-2: aOR, 2.62; 95% CI, 2.06-3.33) and mechanical ventilation. (Figure 3)

## Discussion

In this 8-season, multicenter cross-sectional study of data on more than 26 000 youths hospitalized with ARIs, clinical testing for influenza and RSV changed over time, with a 1.7-fold increase from the winter respiratory illness seasons before the COVID-19 pandemic (2016-2020) to the 2023 to 2024 season. In contrast, clinical testing for SARS-CoV-2 declined from the first season of the pandemic (2020-2021), when there was near-universal testing of 90.4% of youths, to 76.0% during the 2023 to 2024 season. Overall, the frequency of clinical testing for SARS-CoV-2, influenza, and RSV was similar during the 2023 to 2024 season, with approximately three-quarters of hospitalized youths tested for each viral illness. However, even during the 2023 to 2024 season, clinical testing for all 3 viral illnesses varied by key patient characteristics, as youths with ICU hospitalizations and those with underlying medical conditions or prematurity were more likely to be tested.

Multiple factors likely contributed to observed increases in testing for influenza and RSV from before the COVID-19 pandemic to the 2023 to 2024 season. First, the development and increasing use of rapid multiplex point-of-care PCR assays in 2020 for SARS-CoV-2, influenza A and B, and RSV (or quad tests) has likely driven increases in testing for all of these viruses. Multiplex testing provides results for all 4 viruses even if only 1 is clinically suspected. Second, enhanced infection-control practices that encouraged testing for SARS-CoV-2 to reduce viral spread in hospital settings and evolving CDC guidance on testing for SARS-CoV-2, influenza, and RSV to inform hospital infection control, clinical management, and influenza and COVID-19 antiviral treatment during periods of viral cocirculations have likely also contributed to increases in clinical testing.^16,17^ For example, although clinical testing guidance for RSV among hospitalized youths has not changed and routine virologic testing for RSV among hospitalized youths is not recommended, we found that young children with RSV were more likely to receive clinical testing in the 2023 to 2024 season compared with prepandemic seasons.^15^ This finding may result, in part, from incidental RSV results from multiplex testing to guide antiviral treatment decisions for influenza and COVID-19. Although there are few data on how clinical testing for SARS-CoV-2, influenza, and RSV have changed since the COVID-19 pandemic, our findings are consistent with a 2024 analysis of RSV testing practices among infants with bronchiolitis during 2015 to 2023 that found similar testing increases among hospitalized infants over time in 4 US health systems.^11^

During the most recent season of this analysis (2023-2024), three-quarters of all enrolled youths with ARIs had clinical testing for SARS-CoV-2, influenza, and RSV, but youths admitted to the ICU and those with underlying conditions or prematurity were more likely to be tested. These findings suggest that clinicians are more likely to obtain clinical testing to confirm a specific diagnosis in youths with more severe illness and those who are at higher risk of more severe outcomes. For SARS-CoV-2 and influenza, clinical test results may also help guide and confirm antiviral and antibiotic treatment decisions for these high-risk groups. Although prompt empiric treatment without awaiting clinical testing is recommended for youths hospitalized with suspected influenza, studies have identified associations between clinical testing and antiviral use.^18,19^

Findings from this analysis have several implications for interpreting studies based on clinical testing. First, longitudinal analyses spanning more than 2 seasons should be interpreted with caution because the frequency of influenza and RSV testing has changed dramatically from before to after the COVID-19 pandemic, as have characteristics of youths receiving testing (with a greater bias toward testing among youths with critical illness before the pandemic). Second, based on testing patterns during 2020 to 2024, this analysis suggests that 15.6% to 23.4% of COVID-19 infections, 16.7% to 27.3%of influenza infections, and 25.4% to 40.8%of RSV disease infections may be missed when relying solely on clinical testing for case detection and that estimates of disease burden based on clinical testing may require adjustment for underascertainment. Third, youths with critical illness and those with underlying conditions may be overrepresented in COVID-19, influenza, and RSV vaccine effectiveness studies based on clinical testing, even during recent seasons. Fourth, using the proportion positive for SARS-CoV-2, influenza viruses, and RSV among youths with clinical testing to impute numbers of infections among those without clinical testing may be a reasonable approach in most seasons, particularly when most youths have clinical testing, but differences in proportions positive among youths with and without clinical testing do occur.

### Limitations

Several limitations should be considered when interpreting our analysis findings. First, we evaluated testing practices at 6 academic pediatric health systems in the US, and testing practices may not be generalizable to other pediatric health systems or hospital systems that provide inpatient care to youths with acute respiratory illness. However, the geographic breadth of study sites increases the likelihood that national patterns and regional differences were adequately captured. Second, there may be systematic differences in characteristics, such as illness severity, age, or care-seeking behavior, among youths enrolled vs not enrolled. Third, clinical testing may have been overestimated if families who did not have a clinical sample were less likely to participate in the study. Fourth, logistic regression models included in this analysis were adjusted only for study site and do not represent independent factors associated with testing. Fifth, testing practices for COVID-19 and the estimated fraction of COVID-19 infections missed when relying on clinical testing may not represent periods outside the winter respiratory illness season, when SARS-CoV-2 still circulates.

## Conclusions

In this cross-sectional study of 8 respiratory illness seasons from 2016 to 2024, clinical testing for influenza and RSV nearly doubled and bias in testing toward youths with critical illness decreased but was still observed during the 2023 to 2024 season. Testing for COVID-19 was nearly universal during the first season after the pandemic began but has declined in recent seasons. Overall, three-quarters of youths hospitalized with ARIs were tested for SARS-CoV-2, influenza, and RSV as of the 2023 to 2024 season, but a sizeable fraction of infections may be missed when relying solely on clinical testing for case detection. Continued monitoring of clinical testing practices is warranted given that testing practices may continue to evolve. Understanding testing patterns will remain critical to interpreting the increasing number of studies that rely on clinical testing for case identification.

## Notes

Supplement 1. **eTable 1.** Comparison of characteristics between youths who received clinical testing and those who did not for SARS-CoV-2, influenza, and RSV among youths aged <18 years hospitalized with acute respiratory illness, NVSN, 2023-2024, N = 3685 **eTable 2.** Frequency and proportion of hospitalized youths with clinical testing by year and pathogen in the prepandemic season, 2016-2020 **eTable 3.** Clinical respiratory panels by surveillance season and study site, New Vaccine Surveillance Network, 2021-2024 **eTable 4.** Study sites of youths aged <18 years hospitalized with acute respiratory illness by winter respiratory illness season, New Vaccine Surveillance Network (NVSN), 2016-2024, N = 26 073 **eFigure 1.** Study inclusion flow diagram, New Vaccine Surveillance Network, 2016-2024, N = 26 073 **eFigure 2.** RSV clinical testing proportion among hospitalized youths aged <2 years with a discharge diagnosis of bronchiolitis by respiratory season, New Vaccine Surveillance Network, 2016-2024, N = 5385 **eFigure 3.** Clinical testing proportion among hospitalized youths aged <18 years by pathogen, respiratory season, and age group, NVSN, 2016-2024

Supplement 2.
Data Sharing Statement
