# Supplementary material for: Clinical Testing for COVID-19, Influenza, and RSV in Hospitalized Youths, 2016-2024
Source: JAMA Netw Open. 2025 Sep 15;8(9):e2531499. doi: 10.1001/jamanetworkopen.2025.31499 (PMC12439058; doi:10.1001/jamanetworkopen.2025.31499)
Supplement: Supplement 1. — eTable 1. Comparison of characteristics between youths who received clinical testing and those who did not for SARS-CoV-2, influenza, and RSV among youths aged <18 years hospitalized with acute respiratory illness, NVSN, 2023-2024, N = 3685 eTable 2. Frequency and proportion of hospitalized youths with clinical testing by year and pathogen in the prepandemic season, 2016-2020 eTable 3. Clinical respiratory panels by surveillance season and study site, New Vaccine Surveillance Network, 2021-2024 eTable 4. Study sites of youths aged <18 years hospitalized with acute respiratory illness by winter respiratory illness season, New Vaccine Surveillance Network (NVSN), 2016-2024, N = 26 073 eFigure 1. Study inclusion flow diagram, New Vaccine Surveillance Network, 2016-2024, N = 26 073 eFigure 2. RSV clinical testing proportion among hospitalized youths aged <2 years with a discharge diagnosis of bronchiolitis by respiratory season, New Vaccine Surveillance Network, 2016-2024, N = 5385 eFigure 3. Clinical testing proportion among hospitalized youths aged <18 years by pathogen, respiratory season, and age group, NVSN, 2016-2024 [file jamanetwopen-e2531499-s001.pdf]

Supplemental Online Content

Toepfer AP, Rutkowski RE, Sahni LC, et al; for the New Vaccine Surveillance Network Collaborators. Clinical testing for COVID-19, influenza, and RSV in hospitalized youths, 2016-2024. *JAMA Netw Open*. 2025;8(9):e2531499. doi:10.1001/jamanetworkopen.2025.31499

eTable 1. Comparison of characteristics between youths who received clinical testing and those who did not for SARS-CoV-2, influenza, and RSV among youths aged <18 years hospitalized with acute respiratory illness, NVSN, 2023-2024, N = 3685

eTable 2. Frequency and proportion of hospitalized youths with clinical testing by year and pathogen in the prepandemic season, 2016-2020

eTable 3. Clinical respiratory panels by surveillance season and study site, New Vaccine Surveillance Network, 2021-2024

eTable 4. Study sites of youths aged <18 years hospitalized with acute respiratory illness by winter respiratory illness season, New Vaccine Surveillance Network (NVSN), 2016-2024, N = 26 073

eFigure 1. Study inclusion flow diagram, New Vaccine Surveillance Network, 2016-2024, N = 26 073

eFigure 2. RSV clinical testing proportion among hospitalized youths aged <2 years with a discharge diagnosis of bronchiolitis by respiratory season, New Vaccine Surveillance Network, 2016-2024, N = 5385

eFigure 3. Clinical testing proportion among hospitalized youths aged <18 years by pathogen, respiratory season, and age group, NVSN, 2016-2024

This supplemental material has been provided by the authors to give readers additional information about their work.

© 2025 Toepfer AP et al. *JAMA Network Open*.

eTable 1. Comparison of characteristics between those who received clinical testing and those who did not for SARS-CoV-2, influenza, and RSV among youths <18 years old hospitalized with acute respiratory illness[1], NVSN, 2023-2024[2], N = 3,685

Characteristics	**RSV**				**Influenza**				**SARS-CoV-2**			
	Not clinical tested	*Clinical tested*	*aOR (95% CI)*	*p-value*	*Not clinical test*	*Clinical tested*	*aOR (95% CI)*	*p-value*	*Not clinical tested*	*Clinical tested*	*aOR (95% CI)*	*p-value*
	N (%)	*N (%)*			*N (%)*	*N (%)*			*N (%)*	*N (%)*		
**Total Enrolled**	1047	2638	-	-	945	2740	-	-	883	2802	-	-
**Age (months), median (IQR)**	19 (6-64)	20 (6-59)	-	-	20 (6-59)	19 (7-64)	-	-	9 (5-24)	10 (2-20)	-	-
**Age group**												
**0-2 months**	132 (12.6)	376 (14.3)	1.76 (1.28- 2.42)	**0.0004**	131 (13.8)	377 (13.7)	1.35 (0.99- 1.83)	0.055	124 (14.0)	384 (13.7)	1.25 (0.92- 1.72)	0.1488
**3-5 months**	91 (8.7)	198 (7.5)	1.36 (0.95- 1.95)	0.087	85 (8.9)	204 (7.5)	1.13 (0.80- 1.61)	0.4684	78 (8.8)	211 (7.5)	1.11 (0.77- 1.58)	0.5608
**6-11 months**	127 (12.1)	341 (12.9)	1.48 (1.08 2.04)	**0.015**	119 (12.6)	349 (12.7)	1.29 (0.94- 1.77)	0.1042	108 (12.2)	360 (12.9)	1.26 (0.91- 1.73)	0.1594
**12-23 months**	160 (15.3)	446 (16.9)	1.74 (1.29- 2.36)	**0.0003**	140 (14.8)	466 (17.0)	1.59 (1.18- 2.15)	**0.0022**	132 (14.9)	474 (16.9)	1.48 (1.09- 2.01)	**0.0114**
**24-59 months**	205 (19.6)	559 (21.2)	1.51 (1.13- 2.02)	**0.0048**	181 (19.2)	583 (21.3)	1.41 (1.06- 1.88)	0.0178	168 (19.0)	596 (21.3)	1.32 (0.99- 1.78)	0.0561
**5-9 years**	199 (19.0)	462 (17.5)	1.23 (0.92-1.65)	0.153	172 (18.2)	489 (17.8)	1.25 (0.93- 1.67)	0.1294	165 (18.7)	496 (17.7)	1.15 (0.86- 1.55)	0.334
**10-17 years**	133 (12.7)	256 (9.7)	1.0 (ref)	.	117 (12.4)	272 (9.9)	1.0 (ref)	.	108 (12.2)	281 (10.0)	1.0 (ref)	0.334
**Sex**												
**Male**	587 (56.1)	1475 (55.9)	1.0 (ref)	.	526 (55.6)	1536 (56.1)	1.0 (ref)	.	487 (55.2)	1575 (56.2)	1.0 (ref)	.
**Female**	460 (43.9)	1163 (44.1)	0.94 (0.80- 1.10)	0.4851	419 (44.3)	1204 (43.9)	0.95 (0.81- 1.11)	0.5634	396 (44.8)	1227 (43.8)	0.93 (0.79- 1.09)	0.3975
**Race/Ethnicity**												
**White, NH**	414 (39.5)	1104 (41.9)	1.0 (ref)	.	374 (41.2)	1144 (39.6)	1.0 (ref)	.	345 (39.1)	1173 (41.9)	1.0 (ref)	.
**Black, NH**	259 (24.7)	555 (21.0)	1.04 (0.84- 1.29)	0.6927	218 (22.1)	596 (23.1)	1.09 (0.89- 1.35)	0.3756	205 (23.2)	609 (21.7)	1.14 (0.92- 1.41)	0.2292
**Hispanic**	271 (25.8)	727 (27.6)	1.09 (0.88- 1.36)	0.4022	259 (27.1)	739 (27.4)	1.14 (0.92- 1.43)	0.2127	248 (28.1)	750 (26.8)	1.23 (0.98- 1.54)	0.0665
**NH/PI, NH**	4 (0.4)	8 (0.3)	1.22 (0.32- 4.62)	0.7621	4 (0.3)	8 (0.4)	0.96 (0.27- 3.40)	0.9597	2 (0.2)	10 (0.4)	2.25 (0.47- 10.73)	0.3078

© 2025 Toepfer AP et al. *JAMA Network Open*.

**AI/AN, NH**	5 (0.5)	14 (0.5)	0.77 (0.26- 2.29)	0.6443	5 (0.5)	14 (0.5)	0.77 (0.26- 2.28)	0.6406	5 (0.6)	14 (0.5)	0.66 (0.22- 1.96)	0.4649
**Asian, NH**	24 (2.3)	83 (3.2)	1.36 (0.82- 2.26)	0.223	23 (2.9)	84 (2.4)	1.30 (0.79- 2.13)	0.2956	23 (2.6)	84 (3.0)	1.21 (0.74-1.99)	0.4359
**Multirace or Other Nonspecified, NH**	62 (5.9)	130 (4.9)	0.83 (0.57- 1.20)	0.3338	56 (5.2)	136 (5.9)	0.8 (0.56- 1.14)	0.2206	49 (5.6)	143 (5.1)	0.89 (0.61 1.29)	0.5489
**Unknown**	8 (0.7)	17 (0.6)	0.70 (0.26- 1.85)	0.4791	6 (0.7)	19 (0.6)	0.95 (0.35- 2.58)	0.9347	6 (0.7)	19 (0.7)	0.84 (0.31- 2.26)	0.7345
**Insurance status**												
**Public**	638 (60.9)	1483 (56.2)	1.0 (ref)	.	566 (59.9)	1555 (56.7)	1.0 (ref)	.	530 (60.0)	1591 (56.8)	1.0 (ref)	.
**Private**	316 (30.2)	849 (32.2)	1.09 (0.92- 1.30)	0.3001	291 (30.8)	874 (31.9)	1.01 (0.85-1.2)	0.9125	274 (31.0)	891 (31.8)	1.01 (0.85- 1.21)	0.8606
**Public and private**	15 (1.4)	29 (1.1)	1.07 (0.55- 2.07)	0.837	14 (1.5)	30 (1.1)	1.05 (0.54- 2.04)	0.8678	12 (1.4)	32 (1.1)	0.95 (0.47- 1.89)	0.8865
**Self-pay (none)**	71 (6.8)	190 (7.2)	1.00 (0.73- 1.37)	0.973	67 (7.1)	194 (7.1)	0.94 (0.69- 1.28)	0.7255	62 (7.0)	199 (7.1)	1.00 (0.73- 1.37)	0.9683
**Underlying medical conditions [3]**												
**Any underlying condition ≥1**	371 (35.4)	1224 (46.4)	1.53 (1.30- 1.80)	**<.0001**	322 (34.1)	1273 (46.5)	1.63 (1.38- 1.91)	**<.0001**	307 (34.8)	1288 (45.9)	1.56 (1.33- 1.84)	**<.0001**
**Chronic lung condition**	259 (24.7)	748 (28.4)	1.17 (0.98- 1.40)	0.0815	221 (23.4)	786 (28.9)	1.27 (1.06- 1.52)	**0.0078**	208 (23.6)	799 (28.5)	1.26 (1.05- 1.52)	**0.0105**
**Cardiovascular disease**	32 (3.1)	265 (10.1)	3.75 (2.53- 5.56)	**<.0001**	27 (2.8)	270 (9.8)	3.88 (2.57- 5.85)	**<.0001**	34 (3.9)	263 (9.4)	2.73 (1.88- 3.97)	**<.0001**
**Neurologic or neuromuscular disease**	61 (6.0)	303 (11.5)	2.00 (1.48- 2.72)	**<.0001**	54 (5.7)	310 (11.3)	2.09 (1.49- 2.75)	**<.0001**	52 (5.9)	312 (11.1)	1.96 (1.43- 2.68)	**<.0001**
**Immunocompromising condition**	14 (1.3)	97 (3.7)	2.55 (1.43- 4.56)	**0.0014**	14 (1.5)	97 (3.5)	2.46 (1.38- 4.37)	**0.0021**	15 (1.7)	96 (3.4)	2.23 (1.27- 3.91)	**0.0048**
**History of prematurity[4]**	91/510 (17.8)	368/1361 (27.0)	1.64 (1.25- 2.16)	**.0003**	85/475 (17.9)	374/1396 (26.79)	1.50 (1.22- 1.84)	**0.0001**	77/442 (17.4)	382/1429 (26.7)	1.55 (1.25- 1.91)	**<.0001**
**Clinical outcomes**												
**Supplemental oxygen**	609 (58.1)	1662 (63.0)	1.14 (0.97- 1.34)	0.0923	560 (59.3)	1711 (62.5)	1.11 (0.94- 1.30)	0.188	515 (58.3)	1756 (62.7)	1.19 (1.01-1.40)	**0.0336**
**Intensive care unit admission**	89 (8.5)	689 (26.2)	3.21 (2.51- 4.10)	**<.0001**	85 (9.0)	693 (25.4)	3.11 (2.43- 3.98)	**<.0001**	91 (10.3)	687 (24.6)	2.62 (2.06 -3.33)	**<.0001**
**Mechanical ventilation**	12 (1.2)	80 (3.0)	2.85 (1.51- 5.36)	**0.0011**	11 (1.2)	81 (2.9)	2.86 (1.50- 5.45)	**0.0013**	11 (1.3)	81 (2.9)	2.70 (1.42- 5.14)	**0.0024**

1 Acute respiratory illness was defined as at least one of apnea, cough, earache, fever, myalgia, nasal congestion, runny nose, sore throat, vomiting after coughing, shortness of breath, wheezing, or apparent life-threatening event or brief, unexplained but resolved event.

2 Time period was defined as October 1 2023-April 30 2024 (respiratory season)

© 2025 Toepfer AP et al. *JAMA Network Open*.

3 Underlying medical conditions include congenital heart malformation or other heart condition, transplant recipient, cancer, sickle cell anemia, cerebral palsy, seizure disorder or other neurologic or neuromuscular disorder, asthma, reactive airway disease, cystic fibrosis, bronchopulmonary dysplasia, chronic lung disease of prematurity or other chronic lung condition, kidney disease, Down syndrome or other genetic/metabolic disorder, blood disorders, liver disease, diabetes, chronic endocrine condition, chronic gastrointestinal disease, other developmental disabilities.

4 History of prematurity presented among youths less than 2 years old, denominator shown in table.

© 2025 Toepfer AP et al. *JAMA Network Open*.

eTable 2. Frequency and proportion of hospitalized youths with clinical testing[a] , by year and pathogen in the pre-pandemic seasons 2016-2020[b]

Pathogen	2016-2020 n (%)	2016-2017 n (%)	2017-2018 n (%)	2018-2019 n (%)	2019-2020 n (%)
**RSV**	5,072 (42.0)	847 (33.8)	1,193 (36.5)	1,276 (42.7)	1,756 (52.8)
**Influenza Virus**	5,384 (44.5)	852 (34.0)	1,216 (37.2)	1,409 (47.2)	1,907 (57.4)

a Clinical testing was defined as anyone who received an RT-PCR test for one of two viruses (Influenza or RSV) during the hospital visit.
b Time periods were defined as October 1-April 30 (respiratory season) of 2016-2020, with the exception of the 2016-2017 season which began on December 1 when NVSN data collection began.

© 2025 Toepfer AP et al. *JAMA Network Open*.

eTable 3: Clinical respiratory panels by surveillance season and study site, New Vaccine Surveillance Network, 2021-2024*

Surveillance Season	Study site					
	**Vanderbilt**	**Rochester**	**Cincinnati**	**Houston**	**Kansas City**	**Pittsburgh**
2021–2022	BioFire Respiratory Pathogen Panel	Hologic Panther Fusion	LDT	Hologic and GeneXpert	Biofire Respiratory Pathogen Panel Cepheid Xpert xpress Flu/RSV/SARS-CoV2 (2021)	Genmark RVP (RP2 Panel)
2022–2023	BioFire Respiratory Pathogen Panel	Hologic Panther Fusion *[or Focus Simplexa Flu A/B + RSV]*	LDT	Hologic and GeneXpert	BioFire respiratory pathogen panel, FLUVID assay (Flu/RSV/SARS-CoV-2), Abbott assay for RSV and Flu	Cepheid ID or GenMark RP2
2023–2024	BioFire Respiratory Pathogen Panel	Hologic Panther Fusion [or Focus Simplexa Flu A/B + RSV]	LDT	GeneXpert (Flu/RSV/SARS), Panther Fusion (Flu/RSV/SARS, para 1-4, hMPV/adeno/rhino), NeuMoDx (SARS), BioFire Torch (RPP2.1)	BioFire respiratory pathogen panel, FLUVID assay (Flu/RSV/SARS-CoV-2), Abbott assay for RSV and Flu, Hologic Aptima for SARS-CoV-2	Cepheid ID or GenMark RP2

* RVP = Respiratory Virus Panel, LDT = laboratory developed test, EUA = Emergency Use Authorization

© 2025 Toepfer AP et al. *JAMA Network Open*.

eTable 4: Study sites of youths <18 years old hospitalized with acute respiratory illness[a] by winter respiratory illness season[b], New Vaccine Surveillance Network (NVSN), 2016-2024, N=26,073

Characteristic	All		Seasons									
			16-20		20-21		21-22		22-23		23-24	
	N	Col %	N	Col %	N	Col %	N	Col %	N	Col %	N	Col %
**Overall**	26073 (100)	100	12090	100	4388	100	2644	100	3266	100	3685	100
**Study site**												
Nashville	3386	12.9	1822	15.1	622	14.2	167	6.3	356	10.9	419	11.4
Rochester	3061	11.7	1366	11.3	440	10.0	388	14.7	370	11.3	497	13.5
Cincinnati	3104	11.9	1623	13.4	525	11.9	268	10.1	343	10.5	346	9.4
Houston	6988	26.8	2812	23.3	1418	32.3	753	28.5	1041	31.9	964	26.2
Kansas City	2686	10.3	1201	9.9	516	11.8	285	10.7	327	10.0	357	9.7
Pittsburgh	6848	26.3	3267	27.0	867	19.8	783	29.6	829	25.4	1102	29.9

a Acute respiratory illness was defined as at least one of the following: apnea, cough, earache, fever, myalgia, nasal congestion, runny nose, sore throat, vomiting after coughing, shortness of breath, wheezing, or apparent life-threatening event or brief, unexplained but resolved event.

b Time periods were defined as October 1-April 30 (respiratory season) of 2016-2024, with the exception of the 2016-2017 season which began on December 1 when NVSN data collection began and the 2020-2021 season which was defined as October 1, 2020-September 30, 2021, for analyses because of atypical RSV circulation and ongoing SARS-CoV-2 circulation during the summer months of 2021. The 2016-2020 seasons were aggregated and the 2020-2021 season onwards were analyzed individually.

© 2025 Toepfer AP et al. *JAMA Network Open*.

eFigure 1. Study inclusion flow diagram, New Vaccine Surveillance Network, 2016–2024[a], N=26,073

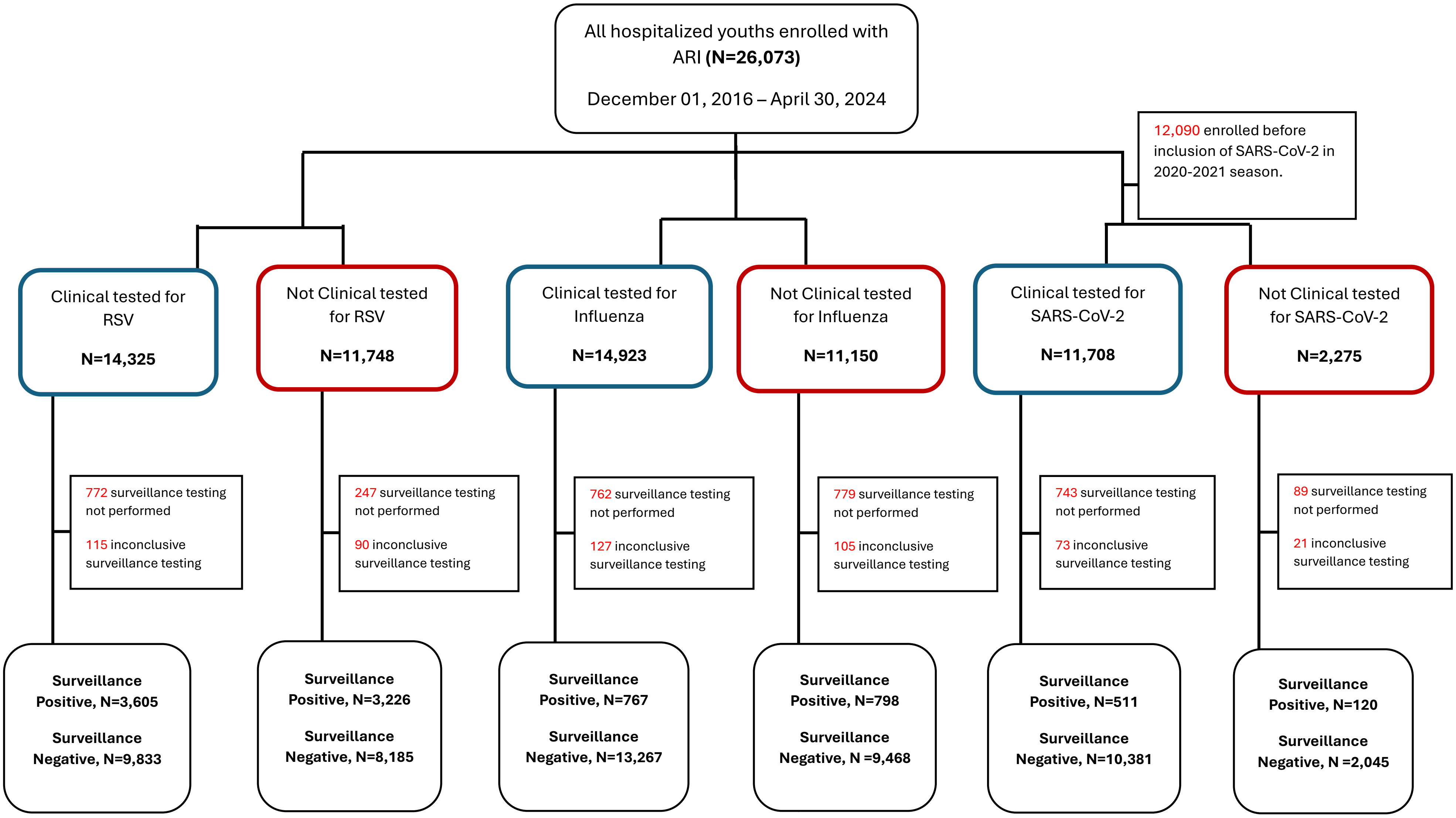

a Time periods were defined as October 1-April 30 (respiratory season) of 2016-2024, with the exception of the 2016-2017 season which began on December 1 when NVSN data collection began and the 2020-2021 season which was defined as October 1, 2020-September 30, 2021, for analyses because of atypical RSV circulation and ongoing SARS-CoV-2 circulation during the summer months of 2021. The 2016-2020 seasons were aggregated and the 2020-2021 season onwards were analyzed individually.

© 2025 Toepfer AP et al. *JAMA Network Open*.

eFigure 2. RSV clinical testing proportion among hospitalized youths <2 years old with a discharge diagnosis of bronchiolitis by respiratory season, New Vaccine Surveillance Network, 2016–2024[a], N = 5,385

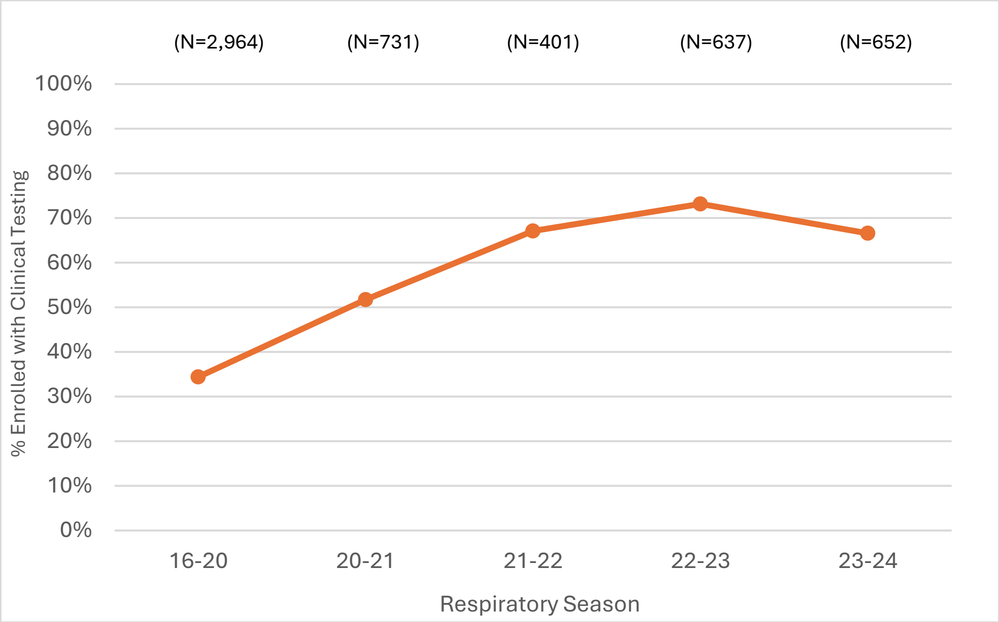

a Time periods were defined as October 1-April 30 (respiratory season) of 2016-2024, with the exception of the 2016-2017 season which began on December 1 when NVSN data collection began and the 2020-2021 season which was defined as October 1, 2020-September 30, 2021, for analyses because of atypical RSV circulation and ongoing SARS-CoV-2 circulation during the summer months of 2021. The 2016-2020 seasons were aggregated and the 2020-2021 season onwards were analyzed individually.

© 2025 Toepfer AP et al. *JAMA Network Open*.

eFigure 3a. SARS-CoV-2 clinical testing proportion among hospitalized youths <18 years old by respiratory season and age group, NVSN, 2016-2024[a]

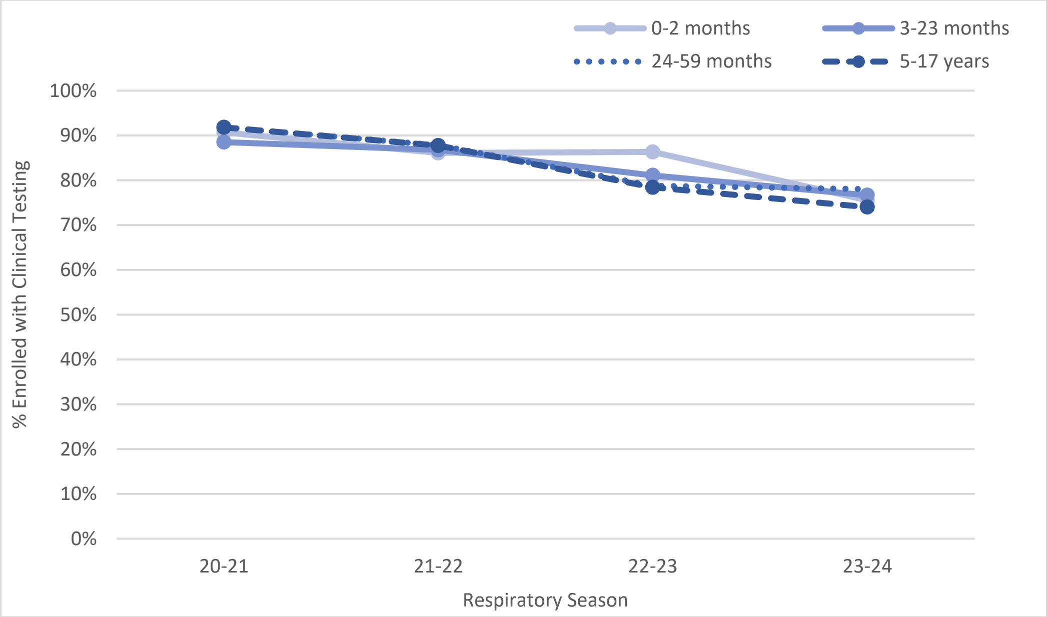

The Cochran-Armitage test for trends for each age grouping was statistically significant ($p<.0001$). Age groups were aggregated to the 4 categories shown because of small numbers in some age strata.

a Time periods were defined as October 1-April 30 (respiratory season) of 2016-2024, with the exception of the 2016-2017 season which began on December 1 when NVSN data collection began and the 2020-2021 season which was defined as October 1, 2020-September 30, 2021, for analyses because of atypical RSV circulation and ongoing SARS-CoV-2 circulation during the summer months of 2021. The 2016-2020 seasons were aggregated and the 2020-2021 season onwards were analyzed individually.

© 2025 Toepfer AP et al. *JAMA Network Open*.

eFigure 3b. Influenza clinical testing proportion among hospitalized youths <18 years old by respiratory season and age group, NVSN, 2016-2024[a]

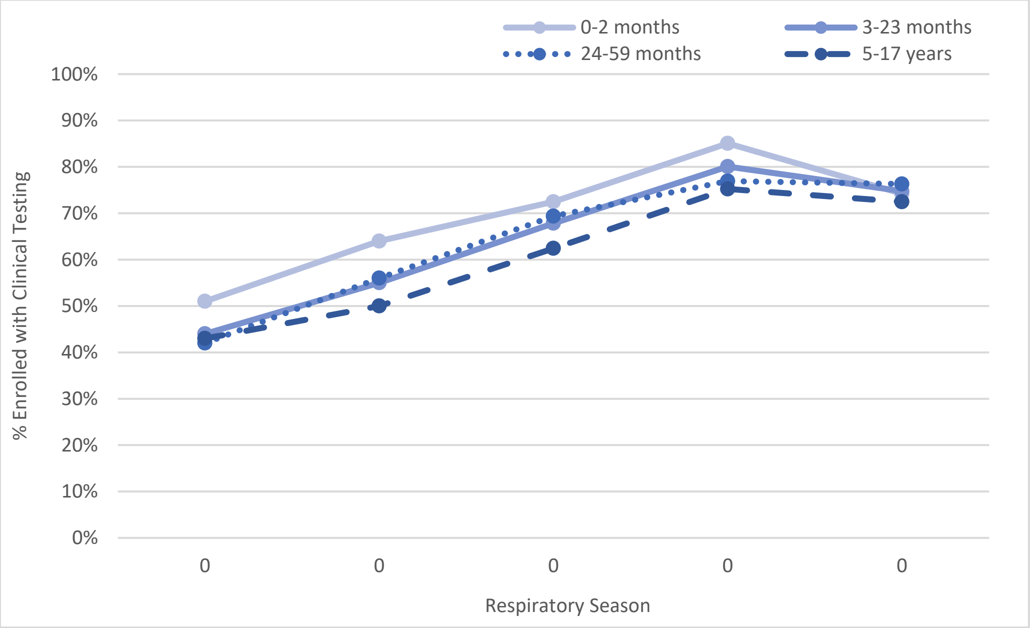

The Cochran-Armitage test for trends for each age grouping was statistically significant ($p<.0001$). The midpoints of each time period were used to represent time in the trend testing to account for unequal spacing. Age groups were aggregated to the 4 categories shown because of small numbers in some age strata.

a Time periods were defined as October 1-April 30 (respiratory season) of 2016-2024, with the exception of the 2016-2017 season which began on December 1 when NVSN data collection began and the 2020-2021 season which was defined as October 1, 2020-September 30, 2021, for analyses because of atypical RSV circulation and ongoing SARS-CoV-2 circulation during the summer months of 2021. The 2016-2020 seasons were aggregated and the 2020-2021 season onwards were analyzed individually.

© 2025 Toepfer AP et al. *JAMA Network Open*.

eFigure 3c. RSV clinical testing proportion among hospitalized youths <18 years old by respiratory season and age group, NVSN, 2016-2024[a]

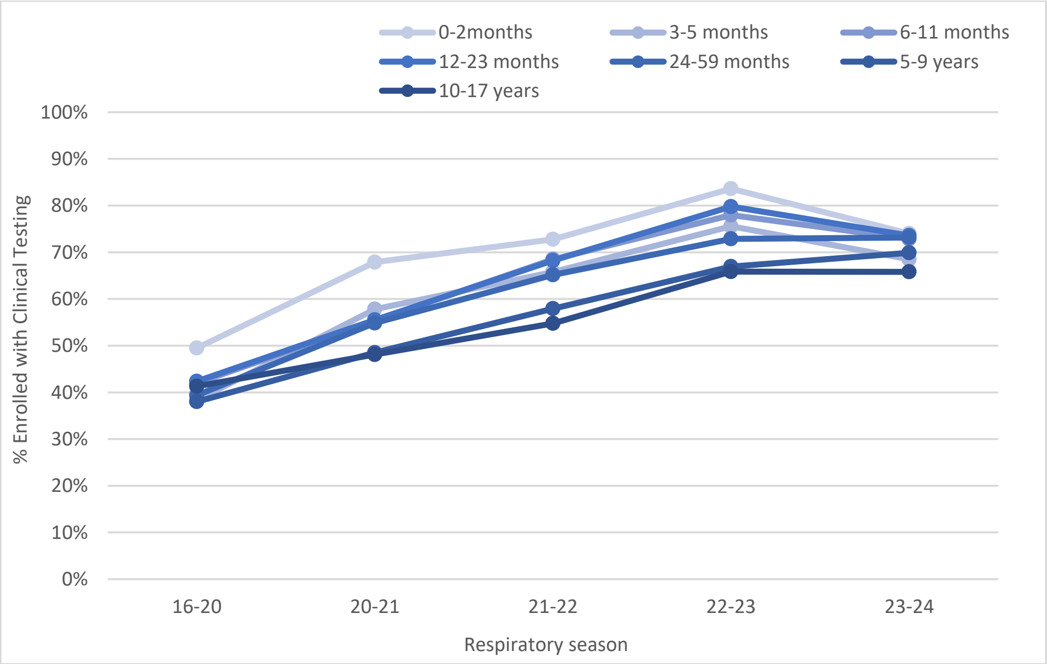

The Cochran-Armitage test for trends for each age grouping was statistically significant ($p<.0001$). The midpoints of each time period were used to represent time in the trend testing to account for unequal spacing.

a Time periods were defined as October 1-April 30 (respiratory season) of 2016-2024, with the exception of the 2016-2017 season which began on December 1 when NVSN data collection began and the 2020-2021 season which was defined as October 1, 2020-September 30, 2021, for analyses because of atypical RSV circulation and ongoing SARS-CoV-2 circulation during the summer months of 2021. The 2016-2020 seasons were aggregated and the 2020-2021 season onwards were analyzed individually.

© 2025 Toepfer AP et al. *JAMA Network Open*.
