# Supplementary material for: Clinical Testing for COVID-19, Influenza, and RSV in Hospitalized Youths, 2016-2024
Source: JAMA Netw Open. 2025 Sep 15;8(9):e2531499. doi: 10.1001/jamanetworkopen.2025.31499 (PMC12439058; doi:10.1001/jamanetworkopen.2025.31499)
Supplement: Supplement 2. — Data Sharing Statement [file jamanetwopen-e2531499-s002.pdf]

Data Sharing Statement

Toepfer. Clinical Testing for COVID-19, Influenza, and RSV in Hospitalized Youths, 2016-2024. *JAMA Netw Open*. Published online September 15, 2025. doi:10.1001/jamanetworkopen.2025.31499

Data
Data available: No
